# Supplementary material for: Identification of photoperiod-induced specific miRNAs in the adrenal glands of Sunite sheep (Ovis aries)
Source: Front Vet Sci. 2022 Jul 22;9:888207. doi: 10.3389/fvets.2022.888207 (PMC9354845; doi:10.3389/fvets.2022.888207)
Supplement: Supplementary file 1 [file Data_Sheet_1.ZIP › Supplementary file/miRDeep2 file.zip!/miRDeep2 file/Know_Structure/LP42A3/oar-mir-26b.pdf]

miRBase precursor : oar-mir-26b
Total read count : 152403
oar-miR-26b read count : 152264
remaining reads : 139

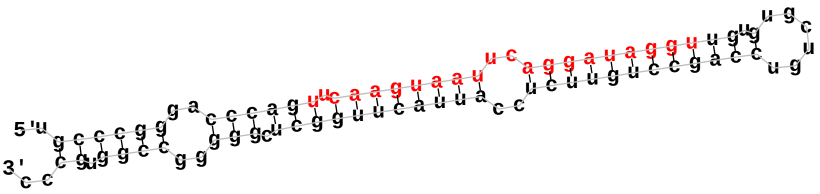

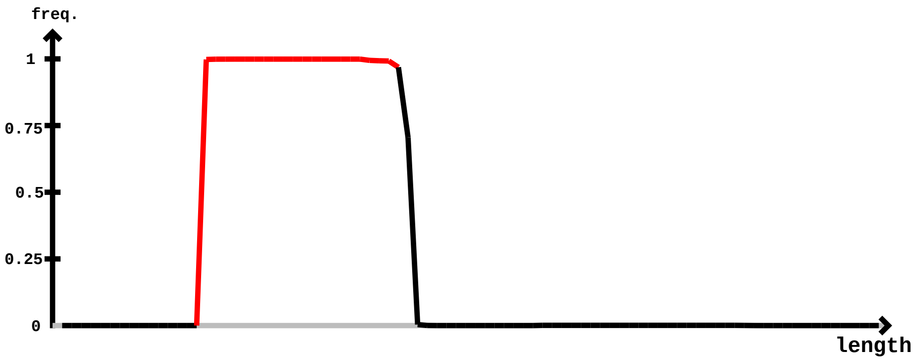


```
                 oar-miR-26b
5'- ugcccgggacccaguucaaguaauucaggauagguugugugcuguccagccuguucuccauuacuuggcucgggggccggugccc   -3'    exp
    .((((((..((((((.((((((((..(((((((((((.(......))))))))))))..))))))))))).))).)))).))..    reads      mm         sample
    .............guucaaguaauucaggauaggu..................................................    2          0          seq
    .............guucaaguaauucaggauagguu.................................................    1          0          seq
    ..............uucaaguaauucaggaG......................................................    9          1          seq
    ..............uucaaguaauucaggaC......................................................    77         1          seq
    ..............uucaaguaauucaggaA......................................................    7          1          seq
    ..............uucCaguaauucaggau......................................................    44         1          seq
    ..............uucaGguaauucaggau......................................................    1          1          seq
    ..............uucaaguaauuUaggau......................................................    1          1          seq
    ..............uucaaguaauucaggau......................................................    559        0          seq
    ..............uucaaguaaCucaggau......................................................    2          1          seq
    ..............Aucaaguaauucaggau......................................................    3          1          seq
    ..............uuUaaguaauucaggau......................................................    2          1          seq
    ..............uucaaguGauucaggau......................................................    1          1          seq
    ..............uucaagCaauucaggau......................................................    1          1          seq
    ..............uucaUguaauucaggau......................................................    1          1          seq
    ..............uCcaaguaauucaggau......................................................    2          1          seq
    ..............uucaaguaauucaggaCa.....................................................    1          1          seq
    ..............uucCaguaauucaggaua.....................................................    14         1          seq
    ..............uucaaguaauucaggaAa.....................................................    3          1          seq
    ..............uucaaguaauucaggaua.....................................................    219        0          seq
    ..............uucaaguaauucaggauag....................................................    131        0          seq
    ..............uucaaguaauucaggauagg...................................................    3572       0          seq
    ..............uucaaguaauucaggauaggu..................................................    39996      0          seq
    ..............uucaaguaauucaggauagguu.................................................    106840     0          seq
    ..............uucaaguaauucaggauagguug................................................    526        0          seq
    ..............uucaaguaauucaggauagguugu...............................................    54         0          seq
    ...............ucaaguaauucaggauagg...................................................    5          0          seq
    ...............ucaaguaauucaggauaggu..................................................    43         0          seq
    ...............ucaaguaauucaggauagguu.................................................    113        0          seq
    ...............ucaaguaauucaggauagguug................................................    3          0          seq
    ................caaguaauucaggauaggu..................................................    2          0          seq
    ................caaguaauucaggauagguu.................................................    20         0          seq
    .................aaguaauucaggauaggu..................................................    2          0          seq
    .................aaguaauucaggauagguu.................................................    6          0          seq
    ...................guaauucaggauagguu.................................................    1          0          seq
```

oar-miR-26b

```
ugcccgggacccaguucaaguaauucaggauagguugugugcuguccagccuguucuccauuacuuggcucgggggccggugccc
..................................uugugugcuguccagccuguuc.............................    1    0    seq
....................................gugugcuguccagccuguucuccauuacuuggcuc..............    2    0    seq
.................................................ccuguucuccauuacuuggc................    5    0    seq
.................................................ccuguucuccauuacuuggcu...............    50   0    seq
.................................................ccuguucuccauuacuuggcuc..............    73   0    seq
.................................................ccuguucuccauuacuuggcucg.............    8    0    seq
```
